# The radiologic roadmap for treatment of an acute appendicitis patient who tested positive for coronavirus disease 19

**DOI:** 10.1259/bjrcr.20210102

**Published:** 2022-03-09

**Authors:** Şeref Barbaros Arik, Elif Gunaydin, Celal İsmail Bİlgiç, İnanç Güvenç

**Affiliations:** 1 Department of Radiology, Medicalpark Hospital, Ankara, Turkey; 2 Department of General Surgery, Medicalpark Hospital, Ankara, Turkey

## Abstract

**Objective:**

In this study, we aimed to emphasize the role of radiological imaging in determining the treatment of a patient, who tested positive for COVID-19 and diagnosed with acute appendicitis during the pandemic.

**Methods:**

A 31-year-old patient presented to the emergency department due to abdominal pain. Ultrasound examination, thoracic and pelvic CT scan were performed.

**Results:**

Non-complicated appendicitis can be treated conservatively with antibiotics. Treatment can be maintained by starting with IV antibiotics and bridging therapy with oral antibiotics.

**Conclusion:**

This studysummarize how radiological follow-up can be used to decide on the suitability of the patient for appropriate medical treatment as an alternative to surgery in a patient, whose gold standard treatment is emergency surgical intervention, which is frequently encountered in the emergency department during the COVID-19 pandemic. Healthcare workers need to be protected to ensure the continuity of the health system. On the other hand, patients requiring emergency healthcare should also be provided with appropriate treatment. Healthcare professionals should choose the most appropriate treatment method, protecting themselves and their patients as much as possible.

## Introduction

Coronavirus Disease 2019 (Covid-19) is a novel contagious disease, which causes severe respiratory failure syndrome. It was first seen in Wuhan, China in December 2019, and was declared as a pandemic by the World Health Organization (WHO) in March 2020 upon spreading to the entire world.^1–3^

The entire world is fighting against this disease. Every day, healthcare professionals publish guidelines and recommendations stating the methods and rules of protection. In the light of these rules and recommendations, the procedures in hospitals are tried to be performed under elective conditions as much as possible and certain procedures are postponed depending on the risks. Despite all these, there may be some inevitable conditions that require emergency intervention.^4,5^

In this study, we aimed to emphasize the role of radiological imaging in determining the roadmap for the treatment of a patient, who tested positive for COVID-19 and diagnosed with acute appendicitis, one of the most common causes of emergency surgical intervention and one of the acute abdominal disorders of general surgery, during the pandemic.

## Case

A 31-year-old patient presented to the emergency department due to abdominal pain. The laboratory tests show high white blood cell count and CRP. There were symptoms of peritoneal irritation in the physical examination notes of the general surgeon.

According to ultrasound (US) examination of the patient who presented to the radiology outpatient clinic, the diameter of the appendix measured as 9 mm. The wall was thick and edematous. There were decreased response to US transducer compression. Adjacent mesenteric fatty tissues were heterogeneous. Findings were consistent with acute appendicitis.

In the preoperative evaluation, COVID-19 test was requested from the patient in line with the recommendations and guidelines, and the result of the test was positive. There were no radiological findings compatible with COVID-19 pneumonia in the thoracic CT of the patient, who did not describe any complaints about the respiratory tract.

**Figure 1.**
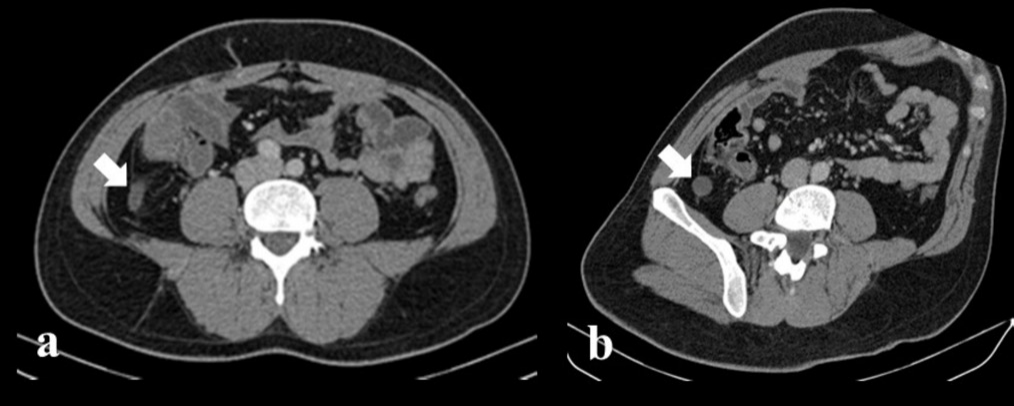
Axial image (**a**) and MPR image (**b**) of enhanced pelvic CT performed after ultrasound. White arrow showed thickened enhanced wall of appendix with increased diameter. No appendicolith detected. Limited mild mesenteric fatty tissue heterogeneity detected in CT, involving only the appendix region, and not extending to the retro peritoneum or pelvic region. No free air and free fluid detected.

Accordingly, pelvic CT with IV contrast was performed to the patient in order to differentiate complicated appendicitis from non-complicated appendicitis. The pelvic CT scan showed findings consistent with appendicitis. No appendicolith was detected. Limited mild mesenteric fatty tissue heterogeneity was detected in CT, involving only the appendix region, and not extending to the retroperitoneum or pelvic region. No free air and free fluid were detected. The diameter of the appendix was measured larger compared to the US examination (Figure 1). It was believed that this was due to the partial compression of the appendix resulting from the pressure applied to the abdomen during US examination, and it was noted as a positive finding.

By consulting with the department of infectious diseases, it was decided that the patient, who was radiologically evaluated as non-complicated appendicitis (Tables 1 and 2), would be quarantined by general surgery until the contagiousness of COVID pneumonia was eliminated, appropriate supportive treatment for Covid-19 would be planned, and conservative non-surgical treatment for appendicitis would be initiated.

**Table 1.** CT Features of Complicated and Non-complicated Appendicitis^6^

CT Findings	Non-complicated Appendicitis	Complicated Appendicitis
Thickened enhanced wall	+	+
Increased diameter	+	+
Periappendiceal stranding	+	+
Appendicolith	+	+
Fluid collection		+
Existence of pus, periappendiceal abscess		+
Necrosis, gangrene		+
Presence of a defect in wall (Perforation)		+
**Presence of extraluminal air (Perforation**)		**+**

CT, Computed tomography.

**Table 2.** Radiological findings and recommendations in symptomatic patients^7^

Radiological Decision	CT Findings	Recommendations
Not appendicitis	<6 mm appendix,>6 mm appendix with a lumen filled with air	Consider other differential diagnoses
Suspected-Uncertain	6–10 mm appendix with no other findings on CT	If it is symptomatic, follow-up is recommended.
Possible appendicitis	6–10 mm appendix +WT + WHE (no FS)	If it is symptomatic, surgery is recommended
Findings consistent with appendicitis	>10 mm apendiks, 6–10 mm apendiks + WT+ WHE+FS	If it is symptomatic, surgery is recommended

FS, Fat stranding; WHE, Wall hyperenhancement; WT, Wall thickening (≥3 mm).

Supportive treatment for Covid-19 ordered by consultant of infectious disease as follows:

1. Favipiravir 1600 mg PO x 2 then 600 mg PO × 2.
2. Paracetamol 500 mg IV × 4.
3. Enoxaparin sodium 4000U IV × 1.
4. Pantoprazole sodium 40 mg IV × 1.

Conservative non-surgical treatment for appendicitis ordered by general surgeon as follows:

1. Oral stop and IV hydration
2. Imipenem&cilastatin 500 mg IV × 4.
3. Metranidazole 500 mg IV × 3.

The patient operated electively after the end of COVID quarantine.

**Figure 2.**
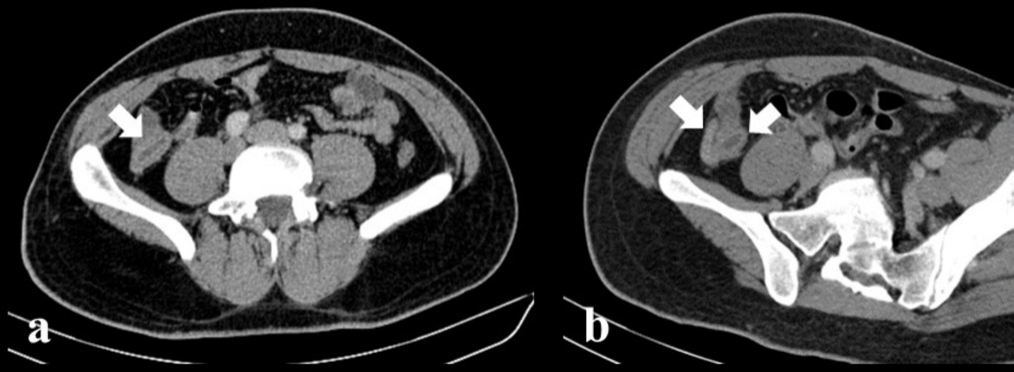
Follow-up axial image (**a**) and MPR image (**b**) of enhanced pelvic CT. White arrow showed appendix. The diameter of the appendix decreased; however, the wall thickness and inflammation continued. There was regression in mesenteric fatty tissue heterogeneity in the periappendicular region. No free air and free fluid detected.

During the follow-up, white blood cell and CRP of the patient decreased. Symptoms and signs regressed. On control CT, it was observed that the diameter of the appendix decreased; however, the wall thickness and inflammation continued. There was regression in mesenteric fatty tissue heterogeneity in the periappendicular region. No free air and free fluid were detected (Figure 2).

## Conclusion

Non-complicated appendicitis can be treated conservatively with antibiotics. Treatment can be maintained by starting with IV antibiotics and bridging therapy with oral antibiotics.^8,9^

In selected cases, the rate of relapses in non-surgical treatments are approximately 12–14%. When the findings of appendicitis spread beyond the right lower quadrant and in the presence of appendicolith, the success of medical treatment is low (failure rate is approximately 30–50%).^4,10^

In appendicitis that is complicated after conservative treatment, percutaneous drainage and catheter placement may be required for abscess formed in the appendix lodge due to interventional radiology.^7^ There is no consensus on an optimal treatment method, preventing operation as a treatment option in complicated cases depending on the decision of the surgeon.^9^

Healthcare workers need to be protected to ensure the continuity of the health system. On the other hand, patients requiring emergency healthcare should also be provided with appropriate treatment. Healthcare professionals should choose the most appropriate treatment method, protecting themselves and their patients as much as possible. This study aims to summarize how radiological follow-up can be used to decide on the suitability of the patient for appropriate medical treatment as an alternative to surgery in a patient, whose gold standard treatment is emergency surgical intervention, which is frequently encountered in the emergency department during the COVID-19 pandemic.

## Learning points

- Radiological imaging can determine the treatment of an acute appendicitis patient in deciding on surgical or conservative treatment.
- Non-complicated appendicitis can be treated conservatively with antibiotics.
- Treatment can be maintained by starting with IV antibiotics and bridging therapy with oral antibiotics.

## References

[1] Romero J, Valencia S, Guerrero A. Acute appendicitis during coronavirus disease 2019 (COVID-19): changes in clinical presentation and CT findings. J Am Coll Radiol 2020; 17: 1011–3. doi: 10.1016/j.jacr.2020.06.002 32610104 PMC7321660

[2] Lu H, Stratton CW, Tang Y-W. Outbreak of pneumonia of unknown etiology in Wuhan, China: the mystery and the miracle. J Med Virol 2020; 92: 401–2. doi: 10.1002/jmv.25678 31950516 PMC7166628

[3] Phelan AL, Katz R, Gostin LO. The novel coronavirus originating in Wuhan, China: challenges for global health governance. JAMA 2020; 323: 709–10. doi: 10.1001/jama.2020.1097 31999307

[4] Gok AFK, Eryılmaz M, Ozmen MM, Alimoglu O, Ertekin C, Kurtoglu MH. Recommendations for trauma and emergency general surgery practice during COVID-19 pandemic. Ulus Travma Acil Cerrahi Derg 2020; 26: 335–42. doi: 10.14744/tjtes.2020.79954 32394416

[5] Wang AW, Prieto J, Ikeda DS, Lewis PR, Benzer EM, Van Gent J-M. Perforated appendicitis: an unintended consequence during the Coronavirus-19 pandemic. Mil Med 2021; 186(1-2): e94–7. doi: 10.1093/milmed/usaa527 33275655 PMC7798865

[6] Foley WD. CT features for complicated versus uncomplicated appendicitis: what is the evidence? Radiology 2018; 287: 116–8. doi: 10.1148/radiol.2018180022 29558303

[7] Pinto Leite N, Pereira JM, Cunha R, Pinto P, Sirlin C. CT evaluation of appendicitis and its complications: imaging techniques and key diagnostic findings. AJR Am J Roentgenol 2005; 185: 406–17. doi: 10.2214/ajr.185.2.01850406 16037513

[8] Turanli S, Kiziltan G. Did the COVID-19 pandemic cause a delay in the diagnosis of acute appendicitis? World J Surg 2021; 45: 18–22. doi: 10.1007/s00268-020-05825-3 33089347 PMC7577362

[9] Talan DA, Saltzman DJ, DeUgarte DA, Moran GJ. Methods of conservative antibiotic treatment of acute uncomplicated appendicitis: a systematic review. J Trauma Acute Care Surg 2019; 86: 722–36. doi: 10.1097/TA.0000000000002137 30516592 PMC6437084

[10] Loftus TJ, Brakenridge SC, Croft CA, Stephen Smith R, Efron PA, Moore FA, et al. Successful nonoperative management of uncomplicated appendicitis: predictors and outcomes. J Surg Res 2018; 222: 212–8. doi: 10.1016/j.jss.2017.10.006 29146455 PMC5742042

